# Tensions between policy and practice: A qualitative analysis of decisions regarding compulsory admission to psychiatric hospital

**DOI:** 10.1016/j.ijlp.2016.02.029

**Published:** 2016

**Authors:** Elizabeth C. Fistein, Isabel C.H. Clare, Marcus Redley, Anthony J. Holland

**Affiliations:** a Education Division, School of Clinical Medicine, University of Cambridge, Cambridge, UK; b National Institute for Health Research Collaboration for Leadership in Applied Health Research and Care East of England (CLAHRC) Cambridge, UK; c Cambridge Intellectual and Developmental Disabilities Research Group, Department of Psychiatry, University of Cambridge, Cambridge, UK; d Cambridgeshire and Peterborough NHS Foundation Trust, Cambridge, UK

**Keywords:** Mental health legislation, Involuntary treatment, Human rights, Autonomy, Paternalism

## Abstract

The use of detention for psychiatric treatment is widespread and sometimes necessary. International human rights law requires a legal framework to safeguard the rights to liberty and personal integrity by preventing arbitrary detention. However, research suggests that extra-legal factors may influence decisions to detain. This article presents observational and interview data to describe how decisions to detain are made in practice in one jurisdiction (England and Wales) where a tension between policy and practice has been described. The analysis shows that practitioners mould the law into ‘practical criteria’ that appear to form a set of operational criteria for identifying cases to which the principle of soft paternalism may be applied. Most practitioners also appear willing, albeit often reluctantly, to depart from their usual reliance on the principle of soft paternalism and authorise detention of people with the capacity to refuse treatment, in order to prevent serious harm. We propose a potential resolution for the tension between policy and practice: two separate legal frameworks to authorise detention, one with a suitable test of capacity, used to enact soft paternalism, and the other to provide legal justification for detention for psychiatric treatment of the small number of people who retain decision-making capacity but nonetheless choose to place others at risk by refusing treatment. This separation of detention powers into two systems, according to the principle that justifies the use of detention would be intellectually coherent, consistent with human rights instruments and, being consistent with the apparent moral sentiments of practitioners, less prone to idiosyncratic interpretations in practice.

## 1 Introduction

The use of compulsory hospital admission for psychiatric assessment and/or treatment is a relatively common practice in many countries (Riecher-Rossler & Rossler, 2007). However, there remains little consensus regarding the circumstances under which it is morally justifiable to use such compulsion, since it deprives the person of their liberty and the legal criteria authorising compulsory admission vary considerably between different jurisdictions (Appelbaum, 1997, Fistein et al., 2009).

International human rights law requires a legal framework to safeguard the rights to liberty and personal integrity of people affected by mental ill-health by preventing arbitrary detention (United Nations, 1991, World Health Organization, 2003). Nonetheless, legal scholars have repeatedly questioned the effectiveness of mental health legislation as a means of protecting the human rights of people receiving psychiatric treatment (Appelbaum, 1997, Gostin, 2008). Empirical research also raises questions regarding the effectiveness of much of this legislation as a safeguard for human rights; for example, rates of detention are not necessarily lower in jurisdictions with stringent legal criteria constraining the use of compulsory admission, nor do they necessarily decrease when a jurisdiction enacts new law with stricter criteria (Zinkler and Priebe, 2002, Salize and Dressing, 2004).

The reasons for this gap between ‘policy’ and ‘practice’ are not fully understood. A body of research based upon clinicians' accounts of their decision-making processes suggests that a complex constellation of factors may influence the decision to detain (Bagby et al., 1991, Engleman et al., 1992, Kullgren et al., 1996, Hoge et al., 1997, Sattar et al., 2006). The role of individual differences in the way risk is assessed by clinicians (Bartlett, 2010) and the role of ‘gut instinct’ based upon professional experience (Glover-Thomas, 2011) have also been highlighted as factors affecting day-to-day mental health decision making. Psychiatrists' accounts of the way in which they learn to make these decisions, through observation of the practice of colleagues, and normally without the benefit of formal training in legal principles, has been cited as an explanation of the discrepancy between policy and practice (Wand & Wand, 2013). However, there is limited recent observational research describing the processes by which actual decisions to admit are made (Holstein, 1988, Quirk et al., 2000).

The aim of this study was to describe the ways in which decisions to detain are made in one jurisdiction (England and Wales) where a tension between policy and practice has been described. We sought to understand the reasons behind day-to-day mental health decision making, to describe the principles on which actual decisions were based, and to analyse how and why they might differ from the legal framework that defines the circumstances under which lawful detention may take place.

In England and Wales, the circumstances under which someone may lawfully be detained in hospital for psychiatric assessment or treatment are defined in the *Mental Health Act 1983 as amended 2007* (MHA). Most compulsory psychiatric admissions are authorised on the grounds given in 2, 3 of the MHA. Two medical practitioners (one of whom has particular expertise in the diagnosis or management of mental disorders) and a specially trained Approved Mental Health Professional (AMHP), who must have a non-medical professional qualification (often, but not necessarily, social work), must agree that the legal criteria for compulsory admission apply.

Section 2 authorises detention in hospital for a period of up to 28 days, for the purpose of assessment. The criteria are that the patient

• *is suffering from mental disorder of a nature or degree which warrants the detention of the patient in a hospital for assessment* (*or for assessment followed by medical treatment*) *for at least a limited period*; *and*
• *he ought to be so detained in the interests of his own health or safety or with a view to the protection of other persons.*

Section 3 authorises detention in hospital for a period of up to six months and can be renewed. The criteria are that the patient

• *is suffering from mental disorder of a nature or degree which makes it appropriate for him to receive medical treatment in a hospital*; and
• *it is necessary for the health or safety of the patient or for the protection of other persons that he should receive such treatment and it cannot be provided unless he is detained under this section*; *and*
• *appropriate medical treatment is available for him.*

The 2007 MHA amendments came into practice in November 2008 and effectively relaxed the criteria for compulsory admission (Glover-Thomas, 2011). These changes were the result of a decade-long debate and were opposed by key stakeholder groups who expressed concerns that the amended Act weakened safeguards for the rights to liberty and self-determination of people at risk of detention (Mental Health Alliance, 2007). In contrast to the mental health legislation of many economically developed countries, there is no requirement to establish that the patient poses a risk to the safety of themselves or others, or that they lack the capacity to make a decision to consent to treatment.

Two years before the amendment of the MHA, the parliament had enacted another new piece of legislation, the *Mental Capacity Act 2005* (MCA), which sets out criteria for the provision of care and treatment (for physical or mental ill-health) deemed necessary in the best interests of people who are unable to give consent, as a result of impairment or dysfunction of mind or brain. In April 2009, additional safeguards concerning in-patient treatment and residential care for people who lack the capacity to give or withhold consent, the MCA Deprivation of Liberty Safeguards (MCA-DoLS) came into force to ensure compliance with Article 5 of the European Convention on Human Rights, as interpreted through a body of case law (Winterwerp v the Netherlands [1979] ECHR, Litwa v Poland [2000] ECHR, HL v UK [2005] ECHR).

The deprivation of liberty is said to occur in circumstances where a person is under continuous control and supervision, is not free to leave, and lacks capacity to consent to these arrangements (*P v Cheshire West and Chester Council and another and P and Q v Surrey County Council* [2014] WLR 2). A deprivation of liberty is lawful only if it represents

• *a proportionate response to the likelihood of* [the patient] *suffering harm and the seriousness of that harm*

and if the person authorizing that restriction

• *reasonably believes that it is necessary* … *in order to prevent harm to* [the patient]

Detention under a MCA DoLS authorisation may be considered less stigmatising, as unlike the MHA there is no connotation with detention for public protection. However, access to independent review and appeal against MCA DoLS authorisation is less straightforward. If a patient objects to the hospitalization or to any of the treatment they will receive there, a MCA DoLS authorisation cannot be granted and detention under the MHA is the only available option.

Consequently, it appeared that the people who decide whether or not to use compulsory admission for psychiatric treatment would be making those decisions within a relatively complex regulatory framework with two key pieces of legislation, one of which potentially conflicted with their professions' values or their personal moral intuitions (Roberts, Peay, & Eastman, 2002). Furthermore, the interface between the two frameworks is complex and poorly understood (Clare et al., 2013, House of Lords Select Committee on the Mental Capacity Act). It remains unclear what the implications of this state of affairs might be for clinical practice.

Understanding the ways in which the new legislation was implemented in practice could potentially highlight the need for specific training or for further law reform. Furthermore, a detailed description of the principles upon which decisions to detain are based in practice, and the way decision makers justify any departure from the legal framework, has broader implications for understanding and addressing the gap between policy and practice that has been observed in multiple studies involving a large number of jurisdictions (Appelbaum, 1997, Zinkler and Priebe, 2002).

## 2 Methods

Over a 12-month period, we collected data on the ways in which decisions to detain people under Section 2 or Section 3 of the MHA were made by medical practitioners and AMHPs working in the catchment area of a mental healthcare provider in the East of England. The study comprised two components:

1) Direct observation of medical practitioners and AMHPs discussing whether adults they had assessed met criteria for compulsory admission and should be detained. These discussions were audio-recorded. In order to assist interpretation, the lead author (EF) also conducted and recorded brief (15–20 min) semi-structured interviews with the medical practitioners and AMHPs immediately after they had made their decisions, asking about the decision-making process.

2) In order to gain a broader understanding of practice than could be obtained through observation of a sample of MHA assessments alone, detailed interviews with medical practitioners and AMHPs, each lasting up to two hours, were also conducted. Following the data collection methods used in Biographic-Narrative Interpretive Methodology (Wengraf, 2001), participants were first asked to tell the story of their involvement with compulsory treatment over the course of their working lives. They were then asked to describe in more detail up to seven particular incidents of decisions to detain that they had mentioned in their stories. This approach was adopted in order to discover the factors that participants consider important when making decisions about detention, rather than imposing an inappropriately narrow, reductionist focus. All interviews were audio-recorded.

Initial sampling was directed towards collecting data from assessments and practitioners that reflected the diversity of situations under which MHA assessments take place, aiming for maximum variability. As the project progressed, an iterative approach was adopted. Data were analysed as they were collected, building understanding and seeking out confirming and disconfirming cases.

A thematic analysis of the data was carried out by EF. All the audio-recordings were transcribed verbatim, followed by initial line-by-line coding. Patterns and relationships between the codes were studied in order to identify themes that arose when participants were making or discussing their decisions about admission to hospital for compulsory assessment or treatment (Braun & Clarke, 2006). This initial analysis was used as the basis for an institutional ethnography (Redley & Weinberg, 2007). Key themes judged particularly relevant to the research question were re-analysed by EF and described in greater detail, paying attention to the ways in which the participants constructed and characterised the importance of concepts such as ‘mental disorder’ or ‘risk’. In order to understand the principles on which participants based their decisions to detain, attention was paid to evidence of responsiveness to proximal interactive cues, evident in the discussions between medical practitioners and AMHPs and to distal institutional mandates, inferred from the researchers' knowledge of the legal frameworks and local policy. To improve the credibility of the conclusions, the analysis was reviewed by IC and AH, who attempted to identify and explore alternative ways of understanding the data.

## 3 Results

During the observational component, seven discussions about the use of compulsory admission or treatment were recorded. These involved fourteen different practitioners: five AMHPs, five psychiatrists, and four primary care physicians (general practitioners or GPs). Three of the seven discussions concerned assessments that had taken place in the patient's home, two had taken place on a mental health in-patient unit, and the remaining two had taken place in the emergency department of a general hospital and in a police custody suite. This is representative of the range of settings where MHA assessments occur. In five of the seven cases, all three practitioners involved in the discussion reached consensus (in two of those cases, the result was detention under the MHA and in the other three cases voluntary treatment was organized, either in hospital or in the community). In the remaining two cases, the medical practitioners recommended detention under the MHA, but the AMHP did not agree that this was necessary and the patient was not detained.

During the interview component, fifteen psychiatrists and one AMHP provided accounts of a further 112 cases (six or seven from each participant) in which a decision about the use of detention was made. Six participants worked in General Psychiatry, five in Older People's Mental Health, two worked with people with Intellectual Disability, one worked in Rehabilitation Psychiatry, one in Liaison Psychiatry, and one in a Forensic Mental Health Team. Five participants were women. Seven participants had experience of mental health practice outside England and Wales. Five participants had more than ten years of professional experience in making decisions about detaining people for compulsory assessment or treatment.

Seven of the sixteen interviewees expressed concerns about the effectiveness of the MHA as a safeguard for the human rights of their patients: three expressed concerns based primarily on the content of the Act, and the other four on the way they perceived the Act being interpreted in practice. One interviewee expressed the view that the Act provided a relatively good safeguard for human rights. The remaining eight interviewees were neutral.

Five key themes emerged from the analysis of the instances and accounts of decision making: (i) diagnosis, (ii) availability of alternatives to detention, (iii) likelihood of response to treatment, (iv) risk assessment, and (v) the patient's capacity to make decisions about treatment. An additional theme that emerged was (vi) the degree of difficulty inherent in the decision making. These themes are discussed in more detail below, accompanied by illustrative excerpts from the data.

### 3.1 Diagnosis

Diagnosis was raised during all of the discussions recorded in the course of the observational component of the study. Before recommending compulsory admission, practitioners explicitly reached a consensus position on the presence of acute mental illness, such as depression, bipolar affective disorder, or schizophrenia. In practice, this limitation of detention to cases of acute mental illness appeared to be achieved in two stages: screening and assessment. All seven assessments observed in this study were arranged following reports of behaviour that could be associated with a severe depressive episode (self-harm, expressions of suicidal ideation) or behaviour that appeared irrational and could be associated with a psychotic episode. At the assessment itself, the purpose of discussing diagnosis appeared to be to establish the presence of acute mental illness, either positively, by gathering evidence of pathognomic symptoms or of pre-existing diagnosis by a trusted authority, or by exclusion of other possibilities:

*Psy4: we even wondered if this was personality disorder …*
*GP3: Mm, I was going to say could it be PD [personality disorder], not depression. PD with alcohol.*
*Psy4: Well this is what we got, you know … But the history didn't support that and {psychotherapist} agreed. You know, the history is admission, ECT, sections, hypomanic spell, you know. It just doesn't fit with a personality disorder. But, you know, in between, reasonable function, but not so much recently.*

This observation was supported by the interview data. Practitioners appeared reluctant to use detention in cases involving long-term conditions judged unlikely to respond to compulsory treatment:

*Sectioning people with dementia, on the whole, is a bad thing, because it's not fair. It's a different deal, getting sectioned if you've got dementia than if you've got functional illness because if you've got functional illness it's likely that with some treatment you will recover and go back to where you were* [*Psy9*]*.*

*And so a lot of people with learning disabilities* [*intellectual disability*] *who'd got in trouble with the law were detained … and one really sometimes struggled to see whether there were genuine benefits that came out of that* [*Psy6*]*.*

### 3.2 Availability of alternatives to detention.

Having agreed upon a diagnosis of mental illness, practitioners discussed whether detention under the MHA was the only appropriate response. The possibility of intensive home treatment (crisis care) was raised and considered as an alternative to hospital admission in all cases where the person being assessed had not already been admitted:

*AMHP1*: *Can she be treated at home*? *This is what I*′*d like to know.*
*Psy2*: *That is*, *I think*, *the big question. There is undoubtedly an element of risk. Can that risk be sufficiently ameliorated in home treatment or not*? *What do you think*?
*Psy1*: *I′d say no. I think, from the little we know, the picture changes a bit too much. An’ I′m not quite sure that home treatment will contain that.*

The AMHP's question about the possibility of home treatment is treated by her colleagues as a call to justify the absence of its use. This suggests that home treatment is considered to be the default option. A lack of availability of round-the-clock crisis care in the community was not raised as a problem by the participants, suggesting that they perceived this to be a readily available alternative to admission despite the fact that lack of its availability has been raised as a factor contributing to the use of detention in some areas (Mind, 2011).

Having established the necessity of in-patient treatment, practitioners discussed the need for compulsory rather than informal admission. A preference for informal admission was a feature of much of the talk; detention was characterised as undesirable whilst informal admission was characterised as the ‘least restrictive option’, to be pursued whenever possible:

*Psy3* [*addressing GP2*]: *We'll recommend a* Section 2 *and the AMHP will complete if she doesn't agree to come in when the ambulance arrives.*
*AMHP2*: *We are guided by the principle of the least restrictive alternative. We want to give her some choice but we also need to keep her safe*…
*Psy3* [*addressing AMHP2*]: *So you said you may decide not to make a recommendation*? *Depending on whether she comes downstairs or not*?
*AMHP2*: *Yes*, *I think that just depends on whether or not*…

The notion of the ‘least restrictive option’ used by practitioners relates to the legal ‘Principle of Less Restriction’, characterised by the requirement within the *MHA Code of Practice* in effect at the time the research was conducted (2008: p5, paragraph 1.3) that ‘*People taking action without a patient's consent must attempt to keep to a minimum the restrictions they impose on the patient's liberty, having regard to the purpose for which the restrictions are imposed*.’ Subsequently, the Code of Practice has been revised and states (Department of Health, 2015) that:

‘*Where it is possible to treat a patient safely and lawfully without detaining them under the Act*, *the patient should not be detained*’ and ‘*If the Act is used*, *detention should be used for the shortest time necessary in the least restrictive hospital setting available*.’

Participants acknowledged that the cases where the patient had been told that she had no choice but to accept hospitalization, could be viewed as wrongful *de facto* detention that sidesteps the procedural safeguards of the MHA: it ‘*bent the rules somewhat in that it was borderline coercion*’ (Psy3) and was ‘*a thinly veiled threat*’ (GP3). In the narrative interviews, five participants described cases where they had difficulty distinguishing the boundary between providing patients with clear information about their options, and introducing an element of coercion that prevented voluntary consent to informal admission. In such cases, the notion of a Principle of Least Restriction was frequently raised as a justification for the decision to attempt to proceed with treatment on an informal basis:

*Psy 1*: *Fortunately*, *he was persuaded to come into hospital voluntarily*, *but that was one very key situation where it felt like we would've been pushed to act.*
*Psy 7*: *We often say we don't want to section people because we're concerned about the therapeutic relationship. Some of that may also be a natural reticence to use a very restrictive practice when we're not by nature policemen.*

### 3.3 Likelihood of response to treatment.

There was very little discussion of the therapeutic *purpose* of hospitalization in the observed assessments, possibly reflecting an implicit assumption that detention would not be considered for any other purpose (a hypothesis supported by the role of diagnosis in limiting consideration of hospitalization to cases of acute mental illness, discussed above). Rather, the practitioners discussed the likely outcomes of the proposed treatment for the patient in question, weighing up the benefits and costs. Detention was treated as unjustifiable if the costs to the patient appeared to outweigh the likely benefits:

*Psy 1*: *Would we bring her into hospital to treat her illness* [schizo-affective disorder] *or would we be just bringing her in as a short term measure to deal with an ongoing problem*, *which is her vulnerability and the difficulties she has with coping given her limitations* … *I quite acknowledge those are concerns*, *but I don't think those are concerns that we're going to sufficiently mediate by bringing her in right now.*
*Psy 2*: *It's not gonna necessarily help her engage with mental health services in the future if we admit her to a psychiatric ward that's likely to be a fairly rough and ready experience. So there are costs to consider to admitting her as well.*

The interview data provide further insights into the intuitions of practitioners regarding the role of the notion of treatability in the justification of detention. Detention was frequently characterised as justifiable in cases where the patient responded well to treatment:

*AMHP 5*: *He* [man detained for treatment for schizophrenia] *went down to a place* [hospital] *in London and actually did really well.*

On the other hand, detention was characterised as difficult to justify when no improvement was anticipated:

*Psy 14*: *It was more and more clear for me that if we detain her* [a teenager with conduct disorder], *it's a very big label on her and it's not at all needed. She does have long-standing issues, but those are all issues that are not something that can be changed, actually, by bringing her into the hospital.*

### 3.4 Risk assessment.

During the observational component of the study, participants were heard discussing three kinds of risk arising from the patient's condition. First, the risks that patients posed to their own safety:

*GP 3*: *So the choices are really do you take the risk of her running off*, *absconding one more time*, *possibly killing herself*, *taking an overdose*, *doing something risky*, *or do you say well look, enough's enough.*

Second, the risks others posed to patients, especially young women patients, all three of whom were deemed to be at risk of ‘exploitation’:

*Psy 3*: *The fact that she was intending to go to university in the state that she is now clearly rendered her not only a danger potentially to other people*, *in terms of her erratic behaviour*, *but certainly to herself and being vulnerable to exploitation and that wasn*'*t acceptable risk for her.*

Third, the risks that the patient posed to others, especially any children living in the household:

*Psy 2*: *She's sleeping very poorly, she's up in the night, she was up at three o'clock in the morning and in with her children. Ah, we just don't know what is … what form her behaviour's going to take.*

Risk assessment also addressed wider notions of ‘best interests’. In two cases, for example, the benefit for the patient of having social and family relationships protected from the deleterious effects of mental illness was discussed:

*Psy 3*: *It was in her best interests according to the legal criteria for her to come in*, *but was also in her best long-term interests for any deterioration or flare-up not to reach the stage where the option to return* [*to the family home*] *would have been precluded.*

This type of discussion could function as a means of acknowledging the views of relatives, which other studies (see, e.g., Kullgren et al., 1996) have identified as a possible influence upon decisions to detain, whilst preserving the principle that the decision to detain should be based upon the interests of the patient.

### 3.5 Decision-making capacity.

The absence of decision-making capacity is not one of the legal criteria for detention under 2, 3 of the MHA. However, during the observational component of the study, the patient's capacity to make decisions about treatment was discussed during all of the cases that resulted in hospitalisation, whether or not the patient was detained under the Mental Health Act. In all of the cases where a recommendation for detention was made, the patient was characterised as unable to understand the nature of their illness and/or the need for treatment. In the only observed case where all the practitioners agreed that informal admission would be appropriate, the patient was characterised as capable of making the decision to accept treatment:

*Psy5* [*addressing his colleagues as they agree to organise a voluntary admission*]: *I think basically he seemed to be willing to come into hospital informally and it seemed that he understood the reasons for the admission. He seemed to be having capacity to make that decision.*

It is striking that the finding of *capacity* occurred in the case of a patient who readily agreed hospital to admission. In all the other cases, the patient had refused hospital admission or asked to be discharged. Medical recommendations for detention were made after patients were characterised as failing to understand the reasons why hospitalisation was being recommended. However, there was no evidence of formal assessment of decision-making capacity. Instead, the absence of capacity appeared to be inferred from failure to agree wholeheartedly to admission or treatment:

*Psy4* [*making the case for detention under the MHA*]: *And you know, there's something almost cognitively lacking in her, in that she'll have, we'll have a long discussion and at the end of it, the ward round, she'll say ‘Can I go home then?’*

This observation raises the possibility that, in practice, incapacity is treated as a necessary condition for justifiable detention, although capacity may be determined on the basis of outcome (i.e., treatment refusals are treated as evidence of incapacity and acceptance of treatment is treated as evidence of capacity) rather than functional ability. This hypothesis is supported by the way that incapacity was defined in the interview data. Most participants characterised treatment refusal as arising from a ‘lack of insight’ and it was this, rather than incapacity as defined in the Mental Capacity Act that, in their opinion, justified detention for compulsory treatment in their eyes:

*Psy6*: *I guess one of the striking things when you move from having spent nearly five years in general medicine into psychiatry, is this idea that not everyone wants to see you, as a patient, and that there is the need for the Mental Health Act … I mean she, in a sense, couldn't see that she was unwell, and therefore why should she accept treatment?*
*Psy17*: *She, of course, had absolutely no insight into the fact that she might have a mental illness. She wouldn't see anyone from mental health services.*

### 3.6 ‘Difficult’ and ‘straightforward’ decisions.

During the interview component of the study, ten participants recounted cases which they described as straightforward decisions. One feature common to the majority of ‘straightforward’ decisions to discharge patients, or not admit them to hospital in the first place, was behaviour that was characterised as an understandable response to a particular social context or environmental trigger rather than a consequence of mental ill-health:

*Psy17*: *So, for example, people have fallen out with their spouses or partners and they've taken an impulsive overdose or made a threatening statement, they have been picked up by the police and when they've spent a couple of hours in the 136 suite or talking to professionals, they calm down and they realise that perhaps yes, things are not going well in the relationship and they're ready to take advice and ask for help. So that's again fairly straightforward, there's not much controversy as to what you do with those things.*

The other common feature in this group of straightforward cases was the emergence of evidence, suggesting that the risk of harm to the patient or others was not as high as it first appeared:

*Psy1*: *I did a general psychiatric assessment, which was just take her history, find out what actually happened, um, explore motives and suicidality, and it transpired that there wasn't very much there at all. She had taken an impulsive overdose. She was not suicidal in mood, she was well supported, she wasn't really depressed, it had all been in response to social stress and she was waiting for her parents to pick her up.*

Features common to the majority of ‘straightforward’ decisions to detain were as follows: (1) an uncontested diagnosis of psychosis or severe mood disorder, (2) a high probability of improvement if treated, (3) impaired decision-making capacity resulting from difficulty understanding the need for treatment (as perceived by the clinicians), (4) a high risk of harm to the patient or others, and (5) the presence of significant distress or disability:

*Psy10*: *He just became very, very psychotic … he thought that he'd cracked some very powerful sort of code and only he knew it. And then he felt that there were these women who were interfering … he actually attacked a girl because of that belief. Not because of anything else, it was because she was interfering with that process that only he was engaged in. And, all of his, like, processes, if you like … to me, he did not have the capacity to make, I think, even small decisions, let alone for his treatment or anything... This was a very unwell man, requires treatment, doesn't have the capacity, and you step in on those grounds.*
*Psy4: But her depression was very convincing, very severe and she was quite disabled by it … And she was probably the most ambivalent lady I′ve ever seen. She would really request an admission, then no sooner had she got to the ward but she would request discharge. And this happened three or four times, and she often would go home and have a relapse and overdose … And I realised that she wasn't in a position, a frame of mind, to make a choice … longitudinally, it became very clear to me that this lady needed to be on section, that she wasn't able to really make these big decisions, and when she made them her suicidality and her depression really sort of confounded the picture and made her change her mind. It was straightforward in that it was very clear to me and her GP, because we had been struggling.*

Cases that appeared to cause particular difficulty included those in which one or more of the features of straightforward cases were not unequivocally present.

Six participants described ‘difficult cases’ where the current degree of mental illness was difficult to assess or the net benefits of admission were difficult to predict. This difficulty was also evident in one of the observed cases. After the assessment of a young woman with a relapsing and remitting psychotic illness, who was also significantly intellectually disadvantaged, the AMHP commented that she had found the decision difficult because:

*AMHP1*: *You have to be convinced that she is so ill she needs to be in hospital in terms of her mental illness. People have seen her before me; they knew what she's like when she's really ill; this was my first acquaintance with her.*

Five participants described ‘difficult cases’ where the two statutory frameworks (the Mental Health Act and the Mental Capacity Act) both appeared applicable, and a decision about which framework to apply was required. This difficulty was also evident in one of the observed discussions:

*Psy2* [*addressing Psy1*]: *I, er, yep. I, I′d confirm you were thinking that she should come into hospital but you're thinking that [AMHP1] might not want to proceed with the application on the grounds that she's consenting to an informal admission?*
*Psy1: Or that she's, yeah, that she's consenting to an informal admission or that she is not actively opposing if there is a doubt about capacity. That's the only thing, I′m just wondering about that point you raised earlier.*
*AMHP1: Yes. But this is the point about Mental Capacity Act, if somebody's going along with it. But then I′m, I myself think that she's, was okay with us, but did say she was not a mental patient earlier on so I′m concerned about…*
*Psy2: And we asked her point blank whether she would consider coming into hospital and to be honest she was, I felt at that point her answer was almost unintelligible.*
*AMHP1: She's not understanding at all. No.*
*Psy2: Due to her thought disorder, due to her illness. And I, in this circumstance, I think this is clearly a circumstance where the Mental Health Act…*
*AMHP1: Takes precedent.*

Finally, six participants described ‘difficult cases’ involving high risks of physical harm. It appears that one complex element of decision-making concerns ideas of responsibility and accountability and their relationship to the level of risk posed by the patient. Practitioners appeared to view themselves as potentially being held accountable for the behaviour of people they did not detain. In most cases, this awareness caused anxiety, and some participants described using detention in cases where they were not completely convinced that it was justified, in order to manage their anxiety:

*Psy17*: *I think I′m being slightly controversial here, but I think the GP's concern here was more about covering our arses for any potential risk, rather than what was in the best interests of the patient. And I was more concerned about the long-term strategy of managing this person, the therapeutic relationship with the team and so on. So I think we all had slightly different takes on what would be the best thing to do in this case. I think eventually, again I′m being a little bit controversial, I think the GP's fears about a potential nasty incident communicated itself sufficiently to both the social worker and me, and we decided the safest option would be for him to be in hospital.*

## 4 Discussion

The results of this study suggest that, when making decisions about the use of compulsory admission, practitioners employed their own ‘practical criteria’. Although the practical criteria are similar to the legal criteria of the Mental Health Act, there are some important differences between policy, as operationalised in mental health legislation, and practice, as enacted in real-life decisions (see Table 1).

In order to analyse the ways in which practitioners justify their decisions, it is useful to consider the underlying principles upon which the legal and practical criteria may be based. Within moral and political philosophy, there are two potential justifications for restricting liberty: the *harm principle*, which states that it may be justifiable to limit someone's liberty in order to prevent them from harming others, and *paternalism* or limitation of someone's liberty for their own good. Whilst the harm principle remains widely accepted as a justification for the deprivation of liberty, paternalism *per se* is more difficult to justify in a liberal system (Mill, 1998[1859]; Feinberg, 1984). However, a limited form, *soft paternalism*, is widely (although not universally) accepted. Soft paternalism justifies limitations on liberty, for the benefit of the person being limited, provided that they are unable to make a choice that would be consistent with their own interests. This lack of decision-making ability may stem from a lack of knowledge about the likely consequences of a particular choice or from an inability to exercise appropriate judgment in making a choice. The degree of capacity required to make a choice, is, according to several influential theorists, on a continuum that varies with the potential consequences of the choice to be made (Feinberg, 1986, Eastman and Hope, 1988, Buchanan and Brock, 1990, McMillan and Gillett, 2005). Thus, a greater degree of interference with liberty may be justifiable in situations where people are at risk of causing themselves significant harm.

The legal criteria for authorising deprivation of liberty under the Mental Capacity Act (through the Deprivation of Liberty Safeguards) appear to provide authorisation on the basis of soft paternalism alone. Patients can only be hospitalized under this framework if they are unable to make their own decision about the need for care and treatment, and if that care and treatment is necessary in their best interests. The legal criteria of the Mental Health Act authorise deprivation of liberty through compulsory admission on the basis of either the harm principle, as patients can be hospitalized if this is necessary for the protection of others, or, alternatively, paternalism as patients can be hospitalized if this is necessary in the interests of their own health or safety. The absence of a test of capacity means that the MHA can be used as legal authorisation for exercising hard paternalism, as a person with a mental disorder who is nonetheless able to make his or her own decision about treatment can still be deprived of liberty in the interests of his or her own health or safety. Many of the misgivings expressed about the amended MHA's effectiveness as a safeguard against unjustifiable deprivation of liberty can be characterised as concerns about its authorisation of hard paternalism, especially when compared with the approach taken by the MCA.

The findings of the analysis of the data appear to form a set of operational criteria for identifying cases where the principle of soft paternalism may be applied. Furthermore, the descriptions of ‘straightforward’ decisions to detain appear to be clear examples of soft paternalism. These observations support a hypothesis that the majority of participants in this study viewed soft paternalism as being both morally permissible and their primary justification for restricting the liberty of their patients. Some practitioners seem to have ‘imported’ the concept of ‘best interests’ from the MCA into their decision making about compulsory admission under the MHA. This is a logical step to take when following the principle of soft paternalism, provided that care is taken to assess the degree to which the paternalism proposed is actually ‘soft’. Without a formal assessment of capacity, and in the context of apparent assumptions that refusal of treatment amounted to absence of insight, which in turn amounted to lack of relevant decision-making capacity, there is a risk that the practitioners would unwittingly make hard paternalistic decisions.

Some of the participants in this study were also prepared to restrict their patients' liberty according to hard paternalism or the harm principle, particularly when the risk of harm to the patient or others appeared significant. However, they did so reluctantly, characterising these decisions as difficult, and suggesting the possibility that, compared with soft paternalism, the harm principle and hard paternalism provide less convincing justifications for detention for psychiatric treatment, at least in the eyes of a group of participants in this study. Some even suggested that they did not view detention in these circumstances as fully justified, but rather action that they felt compelled to take in order to defend themselves against liability for any harm that their patients may cause if not admitted to hospital.

## 5 Strengths and weaknesses.

This study is one of very few direct observational studies into the actual practice of assessing adults for compulsory admission for psychiatric treatment (see also Holstein, 1988 and Quirk et al., 2000). The observational element of the study provided evidence of the way decisions are actually made, not simply the ways in which practitioners describe their decision-making processes after the event. However, MHA assessments are ‘hard to reach’ phenomena and it was not possible to record large numbers of instances of observed decision making. Moreover, it is possible that the presence of an observer recording the decision-making process influenced the outcome of the MHA assessments. However, rates of completion of recommendations for compulsory admissions at observed assessments (72%) were comparable to the rates recorded by the healthcare provider in the 12 months preceding the study (75%), which suggests that the presence of an observer did not have a significant effect on outcome.

Interviews provided rich data about a wide range of instances of decision making, especially in circumstances where observational research would not be practical or appropriate. Triangulation between the two data sources allowed a more comprehensive description of practice than either observation or interviews alone could provide. Whilst a range of medical practitioners were able to participate in interviews, AMHPs, although willing to participate in the observational element of the study, were reluctant to participate in interviews. They cited the impact of their workloads, which is plausible given that, at the time of the study, there were unfilled posts in the AMHP rota. However, the lack of interview data from AMHPs is an unfortunate limitation.

This study provides a detailed description of the principles used for decision making by a particular group of medical practitioners and AMHPs. Further, wider-reaching research is needed to establish whether these principles are widely shared amongst other practitioners, in England and Wales and in other jurisdictions.

## 6 Conclusions

Some aspects of the observed divergence between legal and practical criteria for admission under the MHA may act as an additional safeguard against arbitrary deprivation of liberty. However, other aspects of the divergence may have the opposite effect. The tendency to recommend compulsory admission when this is judged to be in the patient's ‘best interests’, rather than necessary in the interests of his or her health or safety, and the tendency to be swayed towards the use of compulsion in cases where there is judged to be a risk of harm to the patient or others, even when other factors are may be equivocal, may raise the likelihood of compulsory admission. This risk should be addressed in training programmes for practitioners.

One key finding of this study was that some practitioners viewed soft paternalism as the best justification for detention and managed their concern that the MHA authorises detention on other grounds by incorporating an informal test of capacity into their decision making. However, their conceptualisation of capacity as closely related to acceptance of treatment, may, in practice, limit the effectiveness of their test as a safeguard against unjustifiable deprivation of liberty. Once detention is used to safeguard patients' wider ‘best interests’, and not simply in the interests of their health or safety, the possibility of widespread deprivation of liberty opens up unless a coherent and reliable test of capacity is incorporated into the MHA.

Another key finding was that practitioners departed reluctantly from their usual reliance on the principle of soft paternalism to authorise detention when the risk of harm to the patient or others appeared significant. It is possible that the unease expressed by practitioners arises from an awareness that they are stepping outside the usual role of health and social care practitioners to exercise powers usually reserved for the criminal justice system (in the case of enacting the Harm Principle) or powers that are not extended to practitioners treating physical health conditions under the Mental Capacity Act (which does not authorise hard paternalism).

These observations suggest a potential resolution for the tension between policy and practice: replace the problematic MHA and the MCA Deprivation of Liberty Safeguards, which are already acknowledged as requiring reform (House of Lords Select Committee on the Mental Capacity Act 2005, 2014), with two new frameworks, each with a clear function based upon a single theoretical justification for detention.

One framework would provide legal justification for detention on the grounds of soft paternalism. Currently, soft paternalism can be enacted either through the MHA or the MCA DoLS. Patients detained under MCA DoLS have access to fewer procedural safeguards than those detained under the MHA. However, patients detained for the purpose of soft paternalism under the MHA do not have the protection of a functional test of capacity and may be subject to greater stigmatisation due to the connotation of the MHA with public protection. Procedural safeguards could be provided through a relatively informal system of assessments and hearings, similar to those currently in place to ensure that the MHA is used appropriately. With more robust safeguards, a framework justifying detention on the basis of soft paternalism, even in cases where the patient objects, could remain compliant with Article 5 of the European Convention on Human Rights.

The second framework would provide legal justification for detention for treatment needed to reduce the risk of serious harm to others, arising as a result of mental ill-health, for the small number of people who retain decision-making capacity but nonetheless choose to place others at risk by refusing treatment. It could also apply to people who have committed a serious crime as a result of mental ill-health but are more appropriately managed in hospital than in prison. A formal system of judicial oversight could provide procedural safeguards, with the role of medical practitioners reduced to providing information on diagnosis, prognosis and risk management, to facilitate judicial decision making.

This separation of detention powers into two systems, according to the principle which justifies the use of detention would be intellectually coherent, consistent with human rights instruments and, being consistent with the apparent moral sentiments of practitioners, at less risk of idiosyncratic interpretations in practice.

## Figures and Tables

**Table 1:** Legal and practical criteria for compulsory admission and treatment.

Section 3 MHA legal criteria	Practical criteria
The person must have a ‘*disorder or disability of the mind*’ which may encompass conditions such as mental illness, personality disorder or, in the context of ‘*abnormally aggressive or seriously irresponsible conduct*’, intellectual disability.	The person must have a serious mental illness such as a severe depression or an acute psychotic episode.
The disorder must be ‘*of a nature or degree which makes it appropriate for him to receive medical treatment in a hospital* … *and it cannot be provided unless he is detained under this section*’	Compulsory admission must represent the ‘least restrictive alternative’—it must be the case that in-patient treatment is necessary and the patient cannot be persuaded to accept informal admission.
‘*appropriate medical treatment is available*’ defined as: ‘*nursing*, *psychological intervention and specialist mental health habilitation*, *rehabilitation and care*, *the purpose of which is to alleviate*, *or prevent a worsening of*, *the disorder or one or more of its symptoms or manifestations*’	In-patient treatment must be likely to bring about an improvement in the patient's condition, and this benefit must not be outweighed by any risks or disadvantages associated with the treatment plan.
‘*It is necessary for the health or safety of the patient or for the protection of other persons that he should receive such treatment*’	The treatment is in the best interests of the patient, or is necessary to protect others. Treatment is judged to be in the patient's best interests if it protects the patient from harm or exploitation, improves the patient's mental health, or protects the patient's wider interests.
	The patient lacks insight into the diagnosis of mental illness or the need for assessment or treatment.
